# Descriptive Epidemiology of Soil-Transmitted Helminth Infections in the United States: Using Big Data to Characterize Patients and Analyze Parasitic Disease Trends

**DOI:** 10.3390/pathogens13121091

**Published:** 2024-12-10

**Authors:** Chad L. Cross, Bryson Carrier, Miklo A. A. Alcala, Louisa A. Messenger

**Affiliations:** 1 Department of Epidemiology & Biostatistics, School of Public Health, University of Nevada, Las Vegas, NV 89154, USA; 2 Parasitology & Vector Biology (PARAVEC) Laboratory, School of Public Health, University of Nevada, Las Vegas, NV 89154, USA; miklo.alcala@unlv.edu (M.A.A.A.); louisa.messenger@unlv.edu (L.A.M.); 3 Department of Kinesiology and Nutrition Sciences, School of Integrated Health Sciences, University of Nevada, Las Vegas, NV 89154, USA; 4 Department of Environmental & Occupational Health, School of Public Health, University of Nevada, Las Vegas, NV 89154, USA

**Keywords:** parasitology, hookworm disease, ascariasis, trichuriasis, descriptive epidemiology

## Abstract

Soil-transmitted helminths (STH) include species responsible for hookworm disease, ascariasis, and trichuriasis. In the United States, STH infections have been greatly reduced with anthelmintic medications and improved hygiene and sanitation, however, cases still regularly occur, but limited epidemiological data exist. We investigated the occurrence of STH infections using big-data analytics of inpatient medical discharge records (1998–2020). Data were obtained from the Healthcare Cost and Utilization Project National Inpatient Sample. We developed an algorithm to extract International Classification of Diseases codes for STH infections from over 805 million records. We report patient characteristics and other epidemiological data. We found a mean of 223 (SD = 70.1) cases annually over the 23 years. Ascariasis (total *n* = 2599) was the most common, followed by hookworm disease (*n* = 1809) and trichuriasis (*n* = 716). Mean annual cases were highest (*p* < 0.05) in males for hookworm disease (*p* = 0.0313), but equitable for ascariasis and trichuriasis. Age distributions were skewed towards older patients, with whites and Hispanics most common among records. Chronic anemia and heart disease were common comorbidities. This analysis serves as a case study for using patient record databases as a means of indirect parasitic disease surveillance for population-based studies.

## 1. Introduction

Soil-transmitted helminths (STHs) comprise a group of intestinal parasites that spread through contact with contaminated soil, water, or fecal material. These parasites include species responsible for hookworm disease (*Ancylostoma duodenale* and *Necator americanus*), ascariasis (*Ascaris lumbricoides*), and trichuriasis (*Trichuris trichiura*). Though this is not an exhaustive list of potential geohelminthic parasitic infections, these species have traditionally been the focus of investigation in the U.S. owing largely to their historic prevalence [1].

STH infections have a broad distribution, causing tremendous disease burden and problematic medical sequalae globally. STHs are considered a neglected tropical disease, and disproportionally affect those in poverty and/or medically underserved populations [2]. Progress has been made, however, with globally estimated reductions in years lived with disability decreasing through time in hookworm disease (−35.2%), ascariasis (−39.9%), and trichuriasis (−29.3%) from 2007 to 2017 [3]. However, STHs are still widespread and result in millions of cases around the world each year, with the increased use of molecular methods shedding some light on disease prevalence [4]. Unfortunately, the convergence of social, economic, environmental, and political factors results in differential disease burdens at the individual and population levels, which leads to the disparate impact of neglected tropical diseases [5]. 

In the U.S., there is uncertainty about STH prevalence, though it is generally believed that the largest disease burdens are in immigrant populations where these diseases have higher endemicity, or that they may differentially impact medically and demographically marginalized populations, as was historically the case in the rural southeastern U.S. [6,7]. However, little is known about recent STH prevalence owing to the previous successes of anthelminthic drug interventions, improvements in sanitation and hygiene, lack of routine screening, and a predominance of asymptomatic infections. In fact, STHs have been assumed to be mostly eliminated from the U.S. South to the degree that STH infections have not been reportable diseases since the early 1980s [8,9]. Perhaps Lynn et al. captured our current state of knowledge best: “the non-specific nature of clinical illness, the commonality of subclinical disease, and the general lack of clinical suspicion create a scenario in which incidence and prevalence of current infection in the USA are largely unknown and likely undiagnosed” [7] (p. 33). 

The paucity of regular surveillance efforts in the U.S. may lead to the potential for unfortunate medical surprises. Some counties in the southern U.S. are known to be at high risk for continued STH infectivity, with recent reports from the state of Alabama prompting active surveillance in certain counties in the state of Mississippi [8]. 

Our intention in this investigation was to develop a robust indirect and descriptive epidemiological surveillance methodology to understand the prevalence of existing STH infections using available secondary data from hospital patient discharge records.

## 2. Materials and Methods

We sought to understand the potential impact and temporality of STH infections on human health using big-data analytics of inpatient medical discharge records through time (1998–2020). Data beyond 2020 were not yet available at the time of this analysis owing to delayed release and data verification procedures in federal datasets prior to release for use by the research community. Data were obtained from the Agency for Healthcare Research and Quality, the Healthcare Cost and Utilization Project (HCUP), and the Nationwide (1998–2012) and National (2013–2020) Inpatient Sample (NIS), the nation’s largest publicly available inpatient sample [10]. The data represent approximately 35–40 million annual hospitalization discharges. The states providing data have changed through time, but the data from all participating states are drawn for the sample, ultimately representing over 97% of the U.S. population of inpatient medical discharge records [11]. Specifically, a complex-weighted sampling strategy is used to draw a 20% stratified sample from community hospitals in the U.S., which, when weighted properly, can provide unbiased national estimates [12]. Data include many aspects of a patient discharge record, including total charges, human groups of specific ethnicity and sex, and, for some years, hospital census region. Importantly, the data include the International Classification of Disease (ICD Codes), which allow disease conditions of interest to be extracted from the data for analysis. 

ICD codes are particularly valuable for data extraction, as they are standardized across hospitals. The NIS data contain multiple diagnosis columns to allow for more than one disease condition to be recorded for each record. Data from the starting year of 1998 in our study included 15 diagnostic variables; this increased to 33 in later years, and 40 in the most recent year of available data (2020). These changes are related to hospital coding expectations for reimbursement, but also to enhance the ability of hospitals to capture the complexities of numerous diagnoses present in individual patients. Furthermore, database management contracting changes through time as federal contracts change, hence, updated dataset requirements often accompany these contractual change obligations and result in changes to the number of variables collected. Importantly as well, ICD coding requirements changed in the U.S. in 2015. In our data, this means that ICD-9 codes were used from 1998 to 2015 (Quarter 3), and then switched to ICD-10 codes in 2015 (Quarter 4) to the present. The ICD-10 codes provide much more specificity in diagnoses and are useful for understanding subcategories of diseases.

We developed a brute-force extraction algorithm to parse each of our 23 years of data using IBM SPSS (v.29, Armonk, NJ, USA), as our annual databases for NIS are maintained in this software environment. The algorithm scans the entire dataset of the millions of records each year, flags each of the diagnostic columns that have an STH diagnosis code (Table 1), and returns a reduced dataset containing only the discharge records indicating an STH diagnosis. These extracted data are then placed into a data frame for analysis. The full algorithmic approach has been explicated elsewhere for a state-level analysis of cestode diseases using data similar to the NIS [13] (p. 2); additionally, we provide a complete algorithm pseudocode in Appendix A (Algorithm A1).

Because of the complex weighting sampling strategy used to construct the HCUP NIS datasets, the analysis and summarization of the data require the use of complex weights to provide corrected estimates. HCUP provides a discharge weight in each dataset that is used to supply the appropriate epidemiological weight necessary to produce national estimates from the data. All analyses described below utilized epidemiological weights as methodologically described by HCUP to produce correct estimates [14]. 

We investigated STH patient infection discharge data to understand the epidemiological characteristics of STH infections as a surrogate for indirect disease surveillance. We characterized disease cases by type, patient age group, groups of specific ethnicities, total length of hospital stay, and total medical charges. Though the study was largely intended for descriptive epidemiological investigation, we additionally report several summary statistics of these data. In all statistical tests, we report the more robust bootstrapped (*n* = 10,000 resamples) or exact *p*-values in lieu of asymptotic estimates. The following comparisons were made: (1) Temporal Trend: we used the Mann–Kendall test for trend to examine potential monotonic temporal trends in annual case counts; (2) Sex Comparisons: we used independent sample *t*-tests to examine potential differences between sexes in annual counts of each STH; (3) Age Comparisons: we conducted a global analysis of overall differences in annual case counts among age groups using ANOVA followed by Bonferroni-adjusted post-hoc comparisons if the global test was significant; (4) Human Groups of Specific Ethnicity Comparisons: we conducted a global analysis of overall differences in annual case counts among groups of specific ethnicity using ANOVA followed by Bonferroni-adjusted post-hoc comparisons if the global test was significant; (5) Total Charges: we report total charges descriptively using overall weighted mean and accompanying 95% confidence intervals, where weights were represented by annual case count frequencies; (6) Length of Stay: we report hospital length of stay descriptively using overall weighted mean and accompanying 95% confidence intervals, where weights were represented by annual case count frequencies; and (7) Regional Geography: we calculated exact tests of proportions among four census regions for each STH with corrected pairwise proportional tests if overall significance was detected. Unfortunately, state-level estimates cannot be estimated because of the complex-weighting strategy used to compile the NIS data [14]; and (8) Co-Morbidities: we extracted the most common comorbid disease conditions in each STH discharge record to investigate potential co-occurring conditions that may be useful for informing public health outcomes and report them descriptively as percentages. All statistical tests were completed using SAS (v.9.4; SAS, Cary, NC, USA) software.

## 3. Results

The data extraction algorithm we developed scanned over 805 million patient discharge records across the 23 years included in this study. Whereas STH disease conditions were relatively rare each year, a total of 5124 cases were extracted. It is noted that since these cases were from inpatient records, they represent the sickest of the patients, however, they also represent an important proportional number of cases present in the population, most of which are in the outpatient or untreated populations. 

### 3.1. Temporal Trends

**Figure 1:**
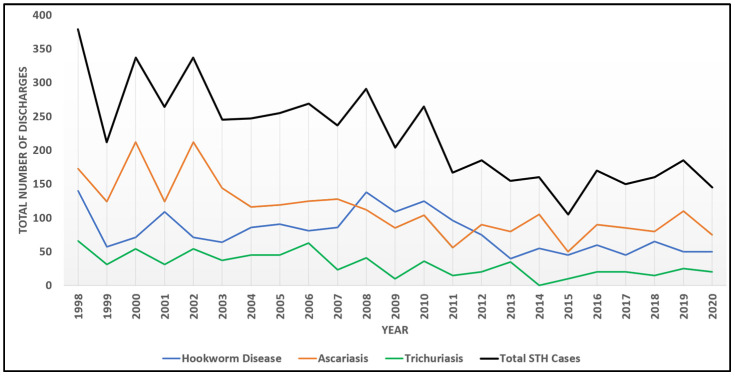
Temporal trends among STH infections from 1998 to 2020.

Among discharges, a mean of 223 (SD = 70.7) cases were found annually. The most common STH was ascariasis (*n* = 2599; 50.7%), followed by hookworm disease (*n* = 1809, 35.3%) and trichuriasis (*n* = 716; 14.0%) for all the years analyzed. Temporal trends were noted among total cases, and displayed evidence of monotonic decrease through time (MK = −0.61, *p* = 0.0004). Significant decreasing trends were also individually present for hookworm disease (MK = −0.34, *p* = 0.0123), ascariasis (MD = −0.61, *p* < 0.0001), and trichuriasis (MK = −0.49, *p* = 0.0019), however, there are notable peaks among diseases with a plateau in overall STH cases beginning in 2016 (Figure 1).

### 3.2. Case Sex Comparisons

We report the results for biological sex utilizing the terminology for this variable as reported in the NIS database, which lists only the binary designation of “female” and “male”. Across the 23 years included in the study, mean annual cases were significantly higher for males compared to females for hookworm disease (t = 2.20, *p* = 0.0313), but no sex differences were found for either ascariasis (t = 1.07, *p* = 0.2891) or trichuriasis (t = 1.66, *p* = 0.1071) (Table 2). Interestingly, though mean case counts did not differ significantly for ascariasis, the total number of cases among females exceeded that of males only for this STH.

### 3.3. Case Age Summary

Cases were categorized into seven age ranges using the standard age groupings provided by the NIS discharge data. Generally, age distributions were heavily skewed towards older patients, with nearly half of the cases in patients 40+ years and combined rates of infection for children under 15 representing 15.79%, 24.28%, and 17.41% for hookworm disease, ascariasis, and trichuriasis, respectively (Table 3). Annual mean cases differed significantly among age groups for hookworm disease (F = 18.43, *p* < 0.0001). Post-hoc corrected *p*-values were significant (*p* < 0.05) for: (1) age group < 1 and age group 1–4 compared to age groups 25–39, 40–64, and 65+; (2) age group 5–14 and age group 15–24 compared to both age groups 40–64 and 65+; and (3) age group 25–39 compared age group 40–64. Annual mean cases also differed significantly among age groups for acariasis (F = 14.18, *p* < 0.0001). Post-hoc corrected *p*-values were significant (*p* < 0.05) for: (1) age group < 1 compared to all older age groups; (2) age group 1–4 compared to 25–39, 40–64, and 65+; and (3) both age group 5–14 and age group 15–24 compared to age group 40–64. Finally, annual mean cases differed significantly among age groups for trichuriasis (F = 7.52, *p* < 0.0001). Post-hoc corrected *p*-values were significant (*p* < 0.05) for both age group < 1 and age group 1–4 compared to age groups 15–24, 40–64, and 65+.

### 3.4. Human Groups of Specific Ethnicity Summary

The NIS discharge database contains a variable labeled as “race/ethnicity” that contains six categories that were used to develop an analysis for human groups of specific ethnicities in this study. We acknowledge that these groupings may differ from commonly accepted inclusive language, but we retain the descriptions that are currently used by HCUP NIS in our summaries, as these are currently utilized in the U.S. electronic health record and are contained in federal reports made from these data. The standardized groupings are white, black, Hispanic, Asian or Pacific Islander, Native American, and Other, the last group of which is used by NIS to characterize any patient who may have indicated a category different from those listed, but the specificity of that selection is otherwise not reported in the database [15]. Patient discharges were majority white for hookworm disease (54.1%), white (30.2%), or Hispanic (30.3%) for ascariasis, and Hispanic (34.6%) for trichuriasis (Table 4). Annual mean cases differed significantly among race/ethnicity groupings for hookworm disease (F = 50.8263, *p* < 0.0001). Post-hoc corrected *p*-values were significant (*p* < 0.05) for white compared to all other groups. Annual mean cases also differed significantly among race/ethnicity groupings for ascariasis (F = 17.01, *p* < 0.0001). Post-hoc corrected *p*-values were significant (*p* < 0.05) for: (1) white compared to Asian or Pacific Islander, Native American, and Other; (2) black compared to Native American; and (3) Hispanic compared to Asian or Pacific Islander, Native American, and Other. Finally, annual mean cases differed significantly among race/ethnicity groupings for trichuriasis (F = 6.66, *p* < 0.0001). Post-hoc corrected *p*-values were significant (*p* < 0.05) for Hispanic compared to Asian or Pacific Islander and Native American.

### 3.5. Case Hospitalization and Charges

The average hospitalization length of stay for STH infections was just over a week (mean = 8.1 days, SD = 2.5 days), with the longest being trichuriasis (mean = 8.7 days, SD = 3.75 days) and shortest hookworm disease (mean = 7.1 days, SD = 2.37 days), with ascariasis intermediate (mean = 8.6 days, SD = 1.87 days), but these did not differ significantly (F = 2.40, *p* = 0.0989). Interestingly, total hospitalization charges were the highest for patient records containing an ascariasis diagnosis (mean = USD 82,313, 95% CI: USD 65,702, USD 98,924), the lowest for records containing a hookworm disease diagnosis (mean = USD 68,141, 95% CI: USD 44,830, USD 91,452), and intermediate for trichuriasis (mean = USD 73,297, 95% CI: USD 50,801, USD 95,792), with a combined STH average of USD 76,102 (95% CI: 74,763, USD 77,441). However, it should be noted that total charges are not specific to treating a single diagnosis (e.g., hookworm disease), but rather include all diagnoses for which a patient is admitted, some of which can be quite medically severe and likewise expensive to treat in hospital, as illustrated when examining comorbid conditions (see Section 3.7 below).

### 3.6. Regional Geography

**Figure 2:**
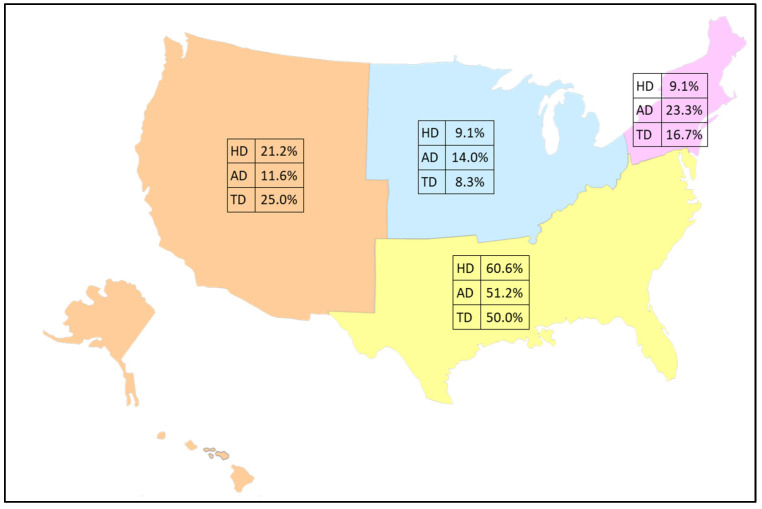
Percentage of discharge records by census region for 2018–2020 among 440 total cases of soil-transmitted helminths (hookworm disease: HD = 165, ascariasis disease: AD = 215, and trichuriasis disease: TD = 60). Base map layer purchased from https://www.editablemaps.com.

Geographic information is not uniformly available across all years of data, making trend comparisons of regional differences not possible. However, regional data were available in the dataset for the final three years of data. The analysis of these records by census reporting regions (i.e., Northeast, Midwest, South, and West) demonstrates that the majority of soil-transmitted helminth infections in inpatient stays are found in the South (Figure 2). The combined prevalence of these infections by region are: Northeast, 75 (17.0%); Midwest, 50 (11.4%); South, 240 (54.5%); and West, 75 (17.0%). Exact tests of homogeneity of proportions with post-hoc corrected proportion pairwise tests indicated significant regional differences for hookworm disease (χ^2^ = 47.48, *p* < 0.0001), with proportions in the south significantly greater than in all other regions (*p* < 0.0001), and rates in the west significantly greater than in the northeast and midwest (*p* = 0.0362). Regional differences in proportions were also found for ascariasis (χ^2^ = 35.57, *p* < 0.0001), with proportions in the south significantly greater than in all other regions (*p* < 0.0001), and the northeast significantly higher than the west (*p* = 0.0279). Finally, regional differences in proportions were found for trichuriasis (χ^2^ = 54.76, *p* < 0.0001), with proportions in the south significantly higher than those in the midwest (*p* = 0.0012) and northeast (*p* = 0.0157).

### 3.7. Comorbid Conditions

Because STH cases were from inpatient hospitalizations, there are many potential patient comorbidities. Hospitalizations rarely occur specifically for a known STH infection, but rather occur owing to a suite of symptoms and laboratory tests that ultimately result in an STH diagnosis appearing in the medical record of already admitted patients. Interestingly, multiple STH infections were not uncommon, with trichuriasis comorbid with hookworm disease, hookworm disease comorbid with ascariasis, and ascariasis comorbid with trichuriasis. Medical conditions consistent with STH infections are seen in the discharge record, with cellulitis of the foot and leg in hookworm disease, volume depletion and electrolyte deficiencies in ascariasis, and bowel issues with comorbid bacterial infections in trichuriasis. Notably, many of these patients were quite ill or had otherwise chronic health problems that would lead to potential hospitalization without any specific admitting diagnosis related to an STH. For example, many had poor heart health, diabetes, kidney failure, and anemia, and demonstrated poor health choices such as tobacco use (Table 5).

## 4. Discussion

STH patient discharges as coded in the NIS database have shown a significant monotonic decline in the United States over the past two decades (Figure 1), which also aligns with reported decreasing global trends in these diseases [3]. Some authors have suggested that these reductions have occurred as a result of interventions targeting hygiene practices in water, sanitation, and hygiene (WASH), as well as targeted public health interventions like mass drug administration (MDA) [16,17,18]. In the U.S., the decrease in hospitalizations associated with STH infections could potentially be attributed to several aspects, including the wide-spread adoption of clean water systems, improved sanitation, and access to anthelmintic treatments, as suggested in a systematic review to assess long-term STH trends in the U.S. However, the authors admit that such speculation is unconfirmed, as there is very limited information about STHs in the U.S. presently [19]. 

Despite apparent case declines across the U.S. as a whole, a plateau in the reduction of STH cases since 2016 requires further exploration and additional years of data to investigate any purported trends more fully. Persistent pockets of infection in impoverished areas indicate that the decline is uneven, with rural and inner-city poverty potentially related to continued STH disease burden in the U.S. [7]. Additional means of surveillance are necessary to parse the overall trends in disease prevalence and to understand differential impacts on certain regions and populations. The current analysis suggests that the southern region of the U.S. has significantly greater burden than other regions, though more years of data are necessary to establish this trend. Historically, the prevalence of hookworm disease in the southern U.S. has been estimated to be over 75% in some states, such as Alabama, and over 65% in Georgia [20,21]. Additionally, ascariasis was postulated to impact Native American children in North Carolina, with a prevalence nearing 50%, and historic prevalence of trichuriasis exceeded an estimated 55% in Kentucky [19]. The acknowledgement of STHs as a long-standing public health concern in the southern U.S. resulted in aggressive intervention by the Rockefeller Sanitary Commission among 11 states in the south, which ultimately reduced STH disease burden in the region, but resulted in erroneous claims of regional eradication of soil transmitted helminths [21]. While public health investment likely played an important role in the reduction of STH disease burden, it is clear from the current analysis that STH infections are still occurring in the region, with state or local-scale research needed to assess current trends at smaller scales than can be assessed with NIS data and that would provide necessary correlates of STH infection to be investigated more thoroughly.

There were some differences found between sexes identified in the analysis. Males were found to have a significantly higher prevalence of hookworm disease compared to females. A recent systematic review has identified male sex as an associated risk factor for some intestinal parasites (e.g., *Strongyloides stercoralis*, *Toxacara canis*, and *T. cati*) in studies conducted in the Appalachian and southern regions of the U.S. [7]. However, data specific for U.S. populations regarding sex differences in the prevalence of hookworm disease have not been largely explored in the recent literature but are suspected to be related to male exposure to environmental contamination while working outdoors [22]. This reasoning has been applied to explain the higher male prevalence in several studies in Asian and African countries, which provide reasonable evidence of higher male prevalence resulting from greater male exposure in contaminated environments [23,24,25,26,27]. Though not statistically significant, females did have a higher number of ascariasis cases compared to males. The higher number may be an artifact of inpatient stays being more common for female patients [28]. Additionally, certain medical conditions that are treated in inpatient settings, such as hepatobiliary and pancreatic conditions associated with ascariasis, are more prevalent among female patients, and hence they may be more likely to be in the inpatient database regardless of STH infection [29,30]. Interestingly, there is recent speculation that differences in sex-mediated immune responses may result in differential infectivity between males and females, with females often having better resistance to infection and better outcomes, and results of future studies in this regard may add an important piece to this puzzle [31]. Additional data to parse our understanding of potential sex-specific risks will allow for targeted health education and prevention strategies to be developed, if needed. 

Unlike in many endemic regions where children are at high risk due to underdeveloped immune systems and frequent exposure to contaminated environments, STH infection case records in the U.S. were more prevalent among older adults [7,16,18]. Data from inpatient medical records show that approximately half of all STH cases occur in individuals aged 40 and above, while combined rates of infection for children under 15 are 15.79%, 24.28%, and 17.41% for hookworm disease, ascariasis, and trichuriasis, respectively. The prevalence of STH infections among all children (15 years and younger) in the U.S. is lower compared to low- and middle-income countries (LMICs), where limited access to clean water, sanitation, and healthcare leads to higher rates of infection [3,32]. For instance, recent studies estimate an overall pooled prevalence of approximately 37.16% among children in LMICs, with regional differences such as a prevalence of 50.41% in the Western Pacific region and 19.86% in the Eastern Mediterranean region [16]. The lower prevalence among children in the database could be attributed to the improved sanitation infrastructure, availability of anthelmintic/antiparasitic drugs, and hygiene practices taught in educational settings that began with targeted public health efforts in the early 20th century resulting from the Rockefeller Sanitary Commission [21]. However, the current prevalence of infection among children in the U.S. is generally unknown because surveillance studies are lacking. Interestingly, a recent study in rural Alabama identified *Necator americanus* infection through qPCR in 42.3% of 26 children tested, though a similar study in Mississippi identified no STHs in a sample of 277 children tested [8,20]. Furthermore, recent studies among U.S. military personnel and their dependent children were likewise heavily skewed towards adult infections compared to children in the study [33]. Certainly, more data are necessary to confirm prevalence among U.S. children. The higher prevalence among older adults may be reflective of the health status at the time of infection, which increased the likelihood of an inpatient stay (discussed later with comorbidities).

Differences in discharge prevalence rates for STHs among human groups of specific ethnicities indicated the highest rates for white patients for hookworm disease. This finding is consistent with historic reports of these infections in the U.S., wherein the prevalence for white patients was 56.1% compared with black patients at 20.3% [34]. In the U.S., there are scant reports of STHs in general, with ethnicity differences largely unknown. The current analysis suggests that white and Hispanic inpatient discharges were equitable for ascariasis diagnosis at 30.2% and 30.3%, respectively, and that trichuriasis was most commonly reported in patient records for Hispanic patients (34.6%), with similar rates among whites (20.0%) and blacks (19.8%). Differences in the frequency of hospital stays for STHs may be unrelated to specific ethnicities in general, however, age-adjusted inpatient hospital utilization among all admitting diagnoses remains equitable among ethnic groups in the U.S. [35], suggesting that differences in STH prevalence among patient records remains an interesting, yet unanswered, question. An additional consideration for future study is the assessment of STH infection among immigrant populations or in travelers coming from regions where infections may be highly endemic [7,36]. Unfortunately, the NIS data do not contain information about patient origin, and this cannot be estimated with the current databases. Analyses at the state or smaller geographic level, if such data are available, may provide additional clues.

As few data are available regarding recent STH infections for U.S. populations, the severity of the infection is also unknown. While this investigation shows that there can be severe cases requiring hospitalization, particularly in individuals with underlying comorbidities, the extent of the infection in the rest of the population remains to be adequately characterized. Common complications associated with STH infections globally are iron deficiency anemia, intestinal obstruction, and chronic malnutrition [16]. The list of the most common comorbidities for hospitalization cases in the U.S. are hypertension, diabetes mellitus (type II), and anemia for combined STH infections. This suggests that the primary cause of hospitalization may not be STH infection, rather the infection is found in the process of in-hospital screening to support treatment for other chronic, non-communicable conditions. This may not hold true for anemia, which may be the primary symptom associated with STH infection requiring hospitalization. Hence, the severity of the infection may be due, in large part, to the health status of the individual at infection onset or due to general health decline as a function of chronic infection. Those that are in poorer health, due to pre-existing conditions, lifestyle choices, etc., may be less able to mount an immune response to the infection, or may be more likely to be hospitalized due to their health status.

The financial burden of hospitalization is significant, particularly for those with complications requiring intensive treatment. The current investigation focuses solely on hospitalization costs, excluding those treated with anthelmintic or antiparasitic drugs by their primary care provider, thus not needing hospitalization. Therefore, the costs analyzed here represent only the most severe cases. However, this does not mitigate the very real burden of treatment costs that amounts to tens of thousands of dollars, as well as missed opportunity costs associated with at least a week of work missed when hospitalized. It is noted that these costs are exacerbated among those who cannot afford adequation outpatient care serviced (i.e., those in poverty or non-/under-insured).

As STH infections are considered by the WHO as NTDs, they are most common in warm, humid climates which create favorable conditions for the survival and development of helminth eggs and larvae [2]. This makes geographic analysis an important factor in STH epidemiology. Based on the known distribution for STHs in the U.S., we would expect to see the greatest number of infections in the South. This is supported by the number of hospitalizations in that region (54.5%). Thus, if future public health programs and surveillance initiatives are to be undertaken in the U.S., the southern U.S. would be a logical place to start [2]. We also suspect that climate change may play an important role in parasite expansion both in the U.S. and globally, particularly among STHs that thrive in warm, moist climates. These range expansions may lead to the increased probability of exposure and, hence, to increased disease burden both among animal and human parasites, hence, future geographic analyses are particularly relevant to understanding the future of STHs in the U.S. and elsewhere [37,38,39,40].

## 5. Conclusions

This analysis serves as a case study for using patient record databases as a means of indirect parasitic disease surveillance. This methodology can be used to improve our understanding of the sociodemographic characteristics of patients most at risk for these infections and may assist in obtaining population-based temporal estimates of parasite disease burden for underfunded public health surveillance programs who lack resources for monitoring disease prevalence.

## Figures and Tables

**Table 1:** International Classification of Disease (ICD) diagnosis codes used to extract diagnoses of soil-transmitted helminths.

Disease Condition	ICD 9 (Through 30 September 2015)	ICD 10 (Beginning 1 October 2015)
**Hookworm Disease**		
Ancylostomiasis	126.0, 126.2, 126.3, 126.8	B76.0
Necatoriasis	126.1	B76.1
Other hookworm diseases	126.9	B76.8
Hookworm disease, unspecified	126.9	B76.9
**Ascariasis**		
Ascariasis with intestinal complications	127.0	B77.0
Ascariasis with other complications		B77.8
Ascariasis pneumonia	127.0 with 484.8	B77.81
Ascariasis with other complications	127.0	B77.89
Ascariasis, unspecified	127.0	B77.9
**Trichuriasis**		
Trichuriasis	127.3	B79

**Table 2:** Soil-transmitted helminth discharge case summaries by sex (1998–2020).

Statistic	Hookworm Disease		Ascariasis		Trichuriasis	
	Male	Female	Male	Female	Male	Female
Total Cases	1044	759	1163	1336	415	297
Mean (Annual)	45.4	33.0	50.6	58.1	18.0	12.9
SD	19.62	18.51	26.73	20.69	12.19	8.50
LCL (95%)	36.91	25.00	39.00	49.14	12.77	9.24
UCL (05%)	53.88	41.00	62.13	67.03	23.31	16.59
Median	38	25	40	57	15	10
Minimum	15	10	10	18	<10 *	<10
Maximum	91	81	119	93	47	29

* Cell sizes <10 cannot be directly reported owing to HCUP NIS data protection rules.

**Table 3:** Soil-transmitted helminth discharge case summaries by age group (1998–2020).

Statistic	Age Group (Years)						
	<1	1–4	5–14	15–24	25–39	40–64	65+
**Hookworm Disease**							
Total Cases	15	90	181	184	354	565	422
%	0.83%	4.97%	9.99%	10.16%	19.55%	31.20%	23.30%
Mean (Annual)	0.7	3.9	7.9	8.0	15.4	24.6	18.3
SD	1.72	4.04	9.26	6.93	7.20	15.92	13.12
LCL (95%)	0	2.16	3.87	5.00	12.28	17.68	12.68
UCL (05%)	1.40	5.66	11.87	11.00	18.51	31.45	24.02
Median	0	5	5	5	18	20	15
Minimum	<10 *	<10	<10	<10	<10	<10	<10
Maximum	<10	11	40	22	29	79	56
Total Cases	15	90	181	184	354	565	422
**Ascariasis Disease**							
Total Cases	23	283	308	322	509	586	497
%	0.91%	11.19%	12.18%	12.74%	20.13%	23.18%	19.66%
Mean (Annual)	1.0	8.5	16.0	17.2	32.0	55.7	40.5
SD	2.37	11.45	11.37	11.10	11.57	12.18	10.02
LCL (95%)	0.00	3.52	11.04	12.38	26.99	50.43	36.18
UCL (05%)	2.05	13.42	20.87	21.98	36.99	60.96	44.85
Median	0	10	10	10	20	25	20
Minimum	<10	<10	<10	<10	<10	<10	<10
Maximum	<10	57	48	36	50	48	39
**Trichuriasis Disease**							
Total Cases	<10	18	102	131	98	203	137
%	0.00%	2.61%	14.80%	19.01%	14.22%	29.46%	19.88%
Mean (Annual)	<10	<10	<10	<10	<10	<10	<10
SD	<10	<10	<10	<10	<10	<10	<10
LCL (95%)	<10	<10	<10	<10	<10	<10	<10
UCL (05%)	<10	<10	<10	<10	<10	12.28	<10
Median	0	0	5	5	0	0	5
Minimum	<10	<10	<10	<10	<10	<10	<10
Maximum	<10	<10	10	19	17	26	21
Total Cases	<10	18	102	131	98	203	137

* Cell sizes <10 cannot be directly reported owing to HCUP NIS data protection rules.

**Table 4:** Soil-transmitted helminth discharge case summaries by age group (1998–2020).

Statistic	Human Group of Specific Ethnicity					
	White	Black	Hispanic	Asian or PI	Native American	Other
**Hookworm Disease**						
Total Cases	895	155	297	121	20	165
%	54.14	9.38	17.97	7.32	1.21	9.98
Mean (Annual)	38.9	6.7	12.9	5.3	0.9	7.2
SD	16.53	5.40	7.80	5.40	3.40	10.46
LCL (95%)	31.76	4.41	9.54	2.92	−0.60	2.65
UCL (05%)	46.06	9.07	16.29	7.60	2.34	11.70
Median	35	5	10	5	0	5
Minimum	20	<10 *	<10	<10	<10	<10
Maximum	89	22	26	22	16	40
Total Cases	895	155	297	121	20	165
**Ascariasis Disease**						
Total Cases	632	405	634	176	45	198
%	30.24	19.38	30.33	8.42	2.15	9.47
Mean (Annual)	27.5	17.6	27.6	7.7	2.0	8.6
SD	9.82	19.79	18.76	5.73	4.47	7.56
LCL (95%)	23.23	9.05	19.45	5.17	0.02	5.34
UCL (05%)	31.73	26.17	35.68	10.13	3.89	11.88
Median	25	15	25	8	0	6
Minimum	10	<10	<10	<10	<10	<10
Maximum	50	99	99	19	20	26
**Trichuriasis Disease**						
Total Cases	115	114	199	86	<10	61
%	20.00	19.83	34.61	14.96	<10	10.61
Mean (Annual)	5.0	5.0	8.7	3.7	<10	2.7
SD	5.04	7.53	7.53	4.51	<10	3.46
LCL (95%)	2.82	1.70	5.40	1.79	<10	1.16
UCL (05%)	7.18	8.21	11.91	5.69	<10	4.15
Median	5	4	10	0	<10	0
Minimum	<10	<10	<10	<10	<10	<10
Maximum	21	32	24	11	<10	12
Total Cases	115	114	199	86	<10	61

* Cell sizes <10 cannot be directly reported owing to HCUP NIS data protection rules.

**Table 5:** Soil-transmitted helminth comorbidities for (1998–2020). The top five comorbid ICD codes are shown along with a list of the comorbidities shared among all STHs. Shared comorbidities are not included within each STH individually but are separated for informational purposes.

CONDITION (STH)	COMORBIDITY
Hookworm Disease (HD)	Cellulitis/abscess of foot (9.4%) Hypopotassemia (9.2%) Cellulitis/abscess of leg (7.6%) Trichuriasis (5.9%) Congestive heart failure (5.3%)
Ascariasis Disease (AD)	Volume depletion (6.9%) Urinary tract infection (6.0%) Hookworm disease (5.1%) Acute pancreatitis (4.7%) Hypopotassemia (4.5%)
Trichuriasis Disease (TD)	Ascariasis (7.9%) Hemorrhoids (6.0%) Volume depletion (5.9%) Acid/base balance disorder (5.6%) *Shigella boydii* infection (5.3%)
Shared Conditions among HD, AD, TD	Essential hypertension (11.9%) Type II diabetes mellitus (5.0%) Anemia, unspecified (5.0%) Iron deficiency anemia (4.9%) Tobacco use disorder (4.6%) Acute kidney failure (4.3%) Congestive heart failure (4.0%) Hyposmolality/Hyponatremia (3.8%)

## Data Availability

The analyses reported herein were for secondary data and were obtained through MOU from HCUP, and may be purchased directly from that source [12].

## Appendix A

Pseudocode for variable extraction as described in Section 2.

**Algorithm A1:** Case Extraction and Summary Analysis Pseudocode Algorithm
***STEP 1: Label any ICD 9/10 code as “1” if present and “0” otherwise and create a set of new temporary variables with the “0/1” indicator.*** **RECODE** Diagnostic columns **IF** ICD code present = 1 **ELSE** ICD code not present = 0 **INTO** temporary disease indicator columns. ***STEP 2: Obtain sum of all recoded variables and create a variable.*** **COMPUTE** SumVariable=sum(across temporary disease columns). ***STEP 3: Recode the new sum variable in “0” if the sum is 0, or “1” if the sum is anything > 0 into a disease diagnosis column.*** **RECODE** SumVariable (0 = 0) (ELSE = 1) **INTO** DiseaseDiag. ***STEP 4: Label the new "0/1" sum variable indicator.*** **VALUE LABELS** DiseaseDiag 0 = ‘Disease absent’ 1 = ‘Disease Present’. ***STEP 5: Give the new sum variable a descriptive label.*** **VARIABLE LABELS** DiseaseDiag ‘Disease descriptive label’. ***STEP 6: Delete temporary variable created in STEP 1.*** **DELETE VARIABLES** temporary disease indicator columns and sum column. ***STEP 7: Format new variable as a binary response column.*** **FORMAT** DiseaseDiag (F1.0). ***STEP 8: Export dataset containing only cases for next processing steps.*** **SAVE** Reduced dataset for DiseaseDiag = 1. ***STEP 9: On reduced dataset, add ICD disease labels for each disease into new disease columns.*** **RECODE** Diagnostic columns (ICD Number String = ICD Number Numeric) **INTO** numeric diagnostic columns. ***STEP 10: Add value labels to ICD numbers.*** **VALUE LABELS** numeric diagnostic columns FOR ALL VARS ICD code = ‘ICD name’. ***STEP 11: Use complex weighting and multiple response computations to obtain frequencies and percentages by key variables of interest.*** **WEIGHT** by discharge weight. **FREQUENCIES** VAR=DiseaseDiag. **MULTIPLE RESPONSE** VARS=numeric diagnostic columns **FOR** categorical vars Sex Age Race Region.
